# A mixed meal tolerance test predicts onset of type 2 diabetes in Southwestern Indigenous adults

**DOI:** 10.1038/s41387-024-00269-3

**Published:** 2024-07-10

**Authors:** Cassie M. Mitchell, Emma J. Stinson, Douglas C. Chang, Jonathan Krakoff

**Affiliations:** 1 Obesity and Diabetes Clinical Research Section, Phoenix Epidemiology and Clinical Research Branch, National Institute of Diabetes and Digestive and Kidney Diseases, National Institutes of Health, Phoenix, AZ USA; 2 Harold Hamm Diabetes Center, University of Oklahoma Health Sciences Center, Oklahoma City, USA

**Keywords:** Physiology, Risk factors

## Abstract

**Background/Objective:**

To identify predictors of incident type 2 diabetes using a mixed meal tolerance test (MMTT).

**Methods:**

Adult Indigenous Americans without diabetes (*n* = 501) from a longitudinal cohort underwent at baseline a 4-h MMTT, measures of body composition, an oral glucose tolerance test, an intravenous glucose tolerance test for acute insulin response (AIR), and a hyperinsulinemic-euglycemic clamp for insulin action (M). Plasma glucose responses from the MMTT were quantified by the total and incremental area under the curve (AUC/iAUC).

**Results:**

At follow-up (median time 9.6 [inter-quartile range: 5.6–13.5] years), 169 participants were diagnosed with diabetes. Unadjusted Cox proportional hazards models, glucose AUC_180-min_ (HR: 1.98, 95% CI: 1.67, 2.34, *p* < 0.0001), AUC_240-min_ (HR: 1.93, 95% CI: 1.62, 2.31, *p* < 0.0001), and iAUC_180-min_ (HR: 1.43, 95% CI: 1.20, 1.71, *p* < 0.0001) were associated with an increased risk of diabetes. After adjustment for covariates (age, sex, body fat percentage, M, AIR, Indigenous American heritage) in three subsequent models, AUC_180-min_ (HR: 1.44, 95% CI: 1.10, 1.88, *p* = 0.007) and AUC_240-min_ (HR: 1.41, 95% CI: 1.09, 1.84, *p* < 0.01) remained associated with increased risk of diabetes.

**Conclusions:**

Glucose responses to a mixed meal predicted the development of type 2 diabetes. This indicates that a mixed nutritional challenge provides important information on disease risk.

**Clinical Trial Registry:**

ClinicalTrials.gov identifier : NCT00340132, NCT00339482

## Introduction

Type 2 diabetes is one of the most prevalent global chronic diseases, and in the United States an estimated 37.3 million people have diabetes, with 90–95% of cases being type 2 [1]. In the last several decades, research has evaluated the impact of nutrition and dietary intake on type 2 diabetes, both as a risk factor and a means of glycemic control [2]. However, the wide variation in individual metabolic responses to diet has prompted the development of individualized weight-loss plans and has subsequently ushered in the era of precision nutrition, which aims to use multi-dimensional data from analytical computing platforms to provide targeted recommendations to an individual based upon a clustering of factors including response to diet and other variables such as genetics, gut microbiome, clinical assays (e.g., lipid profile), and lifestyle measures (e.g., sleep, physical activity) [3–5]. Although the oral glucose tolerance test (OGTT) has been historically the clinical standard for diagnosing and categorizing changes in glucose metabolism, glycemic phenotypes (e.g. impaired fasting glucose versus impaired glucose tolerance), and diabetes, a mixed meal tolerance test (MMTT) is more akin to daily intake as foods are often solid and there is a macronutrient mix of carbohydrates, fats and protein which each uniquely stimulate insulin secretion [4, 6–9]. Compared with an OGTT, an MMTT represents a substantively different nutritional challenge.

Previous research has noted the importance of measuring postprandial glucose and insulin responses to substrates such as lipids to determine individual variability and how that may impact the risk of developing diabetes and disease [10]. Since an MMTT perturbs more systems [3] and may provide more physiologically relevant metabolic responses than an OGTT [9], the utility of an MMTT as a precision nutrition tool is under active investigation. For example, machine-learning models incorporating high-dimensional data have been developed to predict glucose and insulin responses after a mixed meal [5]. In addition, the U.S. National Institutes of Health has recently funded a large multicenter study of approximately 10,000 participants seeking to implement Artificial Intelligence-driven algorithms and dietary response including a single mixed-meal test [11]. The usefulness of these studies is enhanced if postprandial glucose and insulin concentrations provide information about future health outcomes. However, MMTT is not as well established at informing disease risk including the risk of diabetes. Therefore, the aim of this analysis was to assess if glucose and insulin responses to an MMTT can predict the development of type 2 diabetes.

## Methods

### Study design

Individuals who participated in a longitudinal study of the pathogenesis of type 2 diabetes (NCT00339482) [12] and participated in a detailed, inpatient metabolic study assessing determinants of type 2 diabetes (NCT00340132) [13–15] were included in the current analyses. Both studies were approved by the Institutional Review Board of the National Institute of Diabetes and Digestive Kidney Diseases. Prior to participation, volunteers were informed of the nature, purpose, and risks of both studies and written informed consent was obtained from all participants for both studies. Adult participants who were not taking medications known to affect glucose metabolism, did not have type 2 diabetes at baseline, and determined to be healthy based on medical history, physical examination, and routine screening laboratory tests were admitted to our clinical research unit in Phoenix, Arizona from 1982 to 2007. During the inpatient baseline visit participants were administered a weight-maintaining diet (50% carbohydrate, 30% fat, 20% protein) as previously described [16]. The inpatient visit included measurements (described below) of body composition, MMTT, IVGTT, hyperinsulinemic-euglycemic clamp (HIEC), and an OGTT to verify the absence of type 2 diabetes during the baseline inpatient visit. Following the inpatient visit, participants who also participated in the longitudinal study were seen approximately every 2 years for outpatient visits during which an OGTT was performed to assess diabetes status. Diabetes and diagnosis date were determined by OGTT values at the time of the outpatient visit or from a review of medical records [14, 15]. Classification of diabetes was based on the 2003 American Diabetes Association criteria [17].

### Body composition

Body composition was assessed by underwater weighing with determination of residual lung volume by helium dilution or dual-energy X-ray absorptiometry (DXA; DPX-L and Lunar Prodigy, GE Lunar, Madison, WI). Body composition measures were made comparable using previously derived equations [18, 19].

### MMTT

After an overnight fast at 0730 h, participants consumed a standardized breakfast containing 30% of their respective weight-maintaining energy requirements [20, 21]. A subset of participants consumed an additional standardized lunch meal at 1130 h. Meals were composed of 20% protein, 40% carbohydrate, and 40% fat. Blood samples for plasma glucose and insulin were drawn at −15 and 0 min prior to the start of the breakfast meal and thereafter every 30 min for up to 240 minutes. The total and incremental areas under the curve (AUC and iAUC, respectively) for plasma glucose and insulin were calculated with the linear trapezoidal rule [22]. The iAUCs were calculated by subtracting the baseline area from the total AUC. AUC/iAUCs were calculated in two ways (1) 0–180 min and (2) 0–240 min. The peak glucose and insulin values between fasting and 240 min, rise from fasting to peak (Δ = peak – fasting value), and decline from peak to 240 min (Δ = 240 minutes value – peak) were also calculated for each individual.

### OGTT and IVGTT

Participants underwent a 75-g OGTT with venous glucose measurements during the baseline inpatient visit and subsequent outpatient follow-up visits. Acute insulin response (AIR), a measure of insulin secretion, was calculated using 25-g intravenous glucose bolus injected over 3 min and calculated as the mean incremental plasma insulin concentration from the 3rd to 5th minute after injection [15, 21]. The AUC/iAUCs for glucose and insulin during the OGTT were also calculated.

### Hyperinsulinemic-euglycemic clamp (HIEC)

Insulin action was measured using a hyperinsulinemic-euglycemic clamp (HIEC), as previously described [23]. In brief, after an overnight fast, a primed continuous insulin infusion (240 pmol/m^2^/min based on body surface area) was administered for 100 min with a 20% dextrose solution infused at varying rates to maintain a 5.55 mmol/L plasma glucose concentration. The rate of total insulin-stimulated glucose disposal (M) was calculated for the last 40 min of the insulin infusions and corrected for steady-state insulin plasma concentrations and endogenous glucose output. Endogenous glucose output was determined via a primed continuous [3-^3^H] glucose infusion (0.3 µCi/min) prior to (for 120 minutes) and during the insulin infusion. HIEC measures were normalized to estimated metabolic body size (fat-free mass + 17.7 kg) [24].

### Statistical Analyses

Analyses were performed in SAS 9.4 (SAS Institute Inc., Cary, NC, USA). Normally distributed data are presented as mean ± standard error of the mean (SEM), while skewed distributions are reported as median with interquartile range (IQR). Insulin AUC/iAUCs, AIR and M were log transformed to approximate a normal distribution. Surrogate measures of insulin secretion were calculated as ∆ insulin 0 to 30 min/∆ glucose 0 to 30 min and insulin resistance as 1/fasting insulin and multiplied to get the disposition index (DI) [25]. Descriptive statistics of baseline characteristics were compared between participants who were and were not diagnosed with diabetes by the end of the study. Differences between groups (diabetes vs. no diabetes) were assessed using independent sample t-tests for continuous variables and chi-square tests for categorical variables. Paired t-tests were used to compare glucose and insulin AUCs.

Cox proportional hazards models were used to assess the prospective relationship between MMTT glucose and insulin responses and development of diabetes. A series of progressively adjusted models were fit [1]: unadjusted [2]; adjusted for age, sex, body fat (%) [3]; further adjusted for M [4]; further adjusted for AIR and full vs. non-full Southwestern Indigenous American heritage (SWIA). For all insulin measures a final set of models including the corresponding glucose measure were assessed; however, results remained unchanged (results not shown). Proportional hazards assumptions were assessed using Schoenfeld residuals. To facilitate comparisons, all continuous variables were standardized (i.e. mean = 0, SD = 1) and the hazard ratios (HR) were reported per SD.

Pearson correlation coefficients were utilized to assess the bivariate associations between MMTT and OGTT variables. Prediction models of diabetes, accounting for time to event, enabled calculation of C-statistics to compare the predictive abilities of MMTT variables with the corresponding OGTT variable, fasting glucose, 60-min glucose and 120-min glucose from the OGTT [14, 26]. Fasting and 120-min glucose from the OGTT were included as they are known predictors of diabetes. C-statistics were quantified using the Pencina and D’Agostino method and compared using the DeLong method [27, 28]. C-statistics were calculated for significant model 4 measures.

## Results

**Fig. 1:**
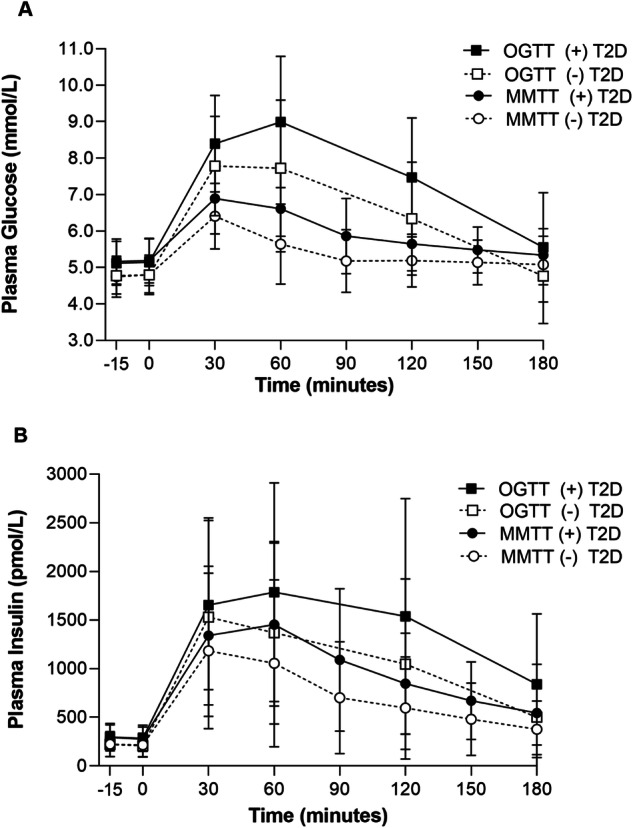
Metabolic response to mixed meal test (MMTT) and oral glucose tolerance test (OGTT). Means and SDs for glucose (**A**) and insulin (**B**) responses by those who developed diabetes and those who did not. MMTT responses are displayed by and closed black circles with solid lines and open circles with dashed lines for participants who developed type 2 diabetes (+T2D) *n* = 169(34%) and those who did not (−T2D) *n* = 332(66%). OGTT responses are displayed similarly with closed and open squares.

Of the 501 participants, 169 (34%) were diagnosed with type 2 diabetes with median follow-up time of 9.6 years (IQR: 5.6–13.5 years). Demographic characteristics are reported in Table 1. Adults who developed type 2 diabetes were more likely to be female, had higher body mass indices (37.0 ± 0.5 kg/m^2^ versus 31.8 ± 0.4 kg/m^2^) and body fat (36.2 ± 0.6% versus 31.2 ± 0.5%). Glucose and insulin responses during the MMTT are shown (Fig. 1A, B). Glucose and insulin AUCs were significantly lower for the MMTT compared with the OGTT (*p*-values < 0.001). At baseline, participants who later developed diabetes had increased glucose and insulin AUCs/iAUCs in response to the MMTT than non-progressors (Table 1).

**Table 1:** Descriptives and baseline physiological markers in adults who did (+) and did not (−) develop Type 2 Diabetes (T2D).

Variable	Total	( + ) T2D	(−) T2D
*Demographics*			
*n* (%)	501	169 (34%)	332 (66%)
*Sex, n (%)*			
Male	279 (56%)	81 (48%)	198 (60%)*
Female	222 (44%)	88 (52%)	134 (40%)
*SWIA, n (%)*			
Full	374 (75%)	142 (84%)	232 (70%)**
Other	127 (25%)	27 (16%)	100 (30%)
Age (years)	26.5 (0.3)	26.9 (0.5)	26.4 (0.4)
Follow-up time (years)^a^	9.6 (5.6–13.5)	8.5 (5.0–12.2)	10.1 (6.3–13.9)*
*Body composition measurements*			
BMI (kg/m^2^)	33.5 (0.3)	37.0 (0.5)	31.8 (0.4)**
Weight (kg)	92.4 (1.0)	101.3 (1.7)	87.9 (1.1)**
Height (cm)	166.0 (0.4)	165.3 (0.7)	166.4 (0.4)
Fat mass (kg)	31.4 (0.6)	37.3 (1.0)	28.4 (0.7)**
Fat-free mass (kg)	61.0 (0.6)	64.0 (1.0)	59.6 (0.7)**
Body Fat (%)	32.9 (0.4)	36.2 (0.6)	31.2 (0.5)**
*IVGTT*			
AIR (pmol/L)^a^	1320 (843–2005)	1186 (688–1853)	1376 (921–2064)*
*Hyperinsulinemic-euglycemic clamp*			
M (mg/kg EMBS/min)^a^	2.4 (2.0–3.0)	2.1 (1.9–2.5)	2.6 (2.1–3.2)**
*OGTT*			
Fasting plasma glucose (mmol/L)	4.9 (0.02)	5.1 (0.05)	4.8 (0.03)**
2-h plasma glucose (mmol/L)	6.7 (0.07)	7.5 (0.1)	6.3 (0.1)**
Total_180-min_ glucose AUC (mmol/L×180)	1233.3 (9.9)	1346.8 (16.0)	1175.5 (11.3)**
Incremental_180-min_ glucose AUC (mmol/L×180)	351 (8.2)	423.5 (13.7)	314.0 (9.7)**
Fasting plasma insulin (pmol/L)	240.6 (6.0)	290.5 (10.5)	215.2 (6.4)**
2-h plasma insulin (pmol/L)	1213.4 (46.4)	1537.7 (93.8)	1046.7 (48.6)**
Total_180-min_ insulin AUC (pmol/L×180)	209672.7 (6054.8)	251832.5 (11671.2)	188009.1 (6631.6)**
Incremental_180-min_ insulin AUC (pmol/L×180)	166343.1 (5353.3)	299551.6 (10568.6)	149279.0 (5804.5)**
*Mixed Meal Test*			
Fasting plasma glucose (mmol/L)	4.9 (0.03)	5.2 (0.05)	4.8 (0.03)**
Total_180-min_ glucose AUC (mmol/L×180)	999.3 (6.0)	1066.2 (10.1)	966.7 (6.8)**
Total_240-min_ glucose AUC (mmol/L×240)	1305.4 (6.9)	1379.9 (11.7)	1268.9 (7.8)**
Incremental_180-min_ glucose AUC (mmol/L×180)	116.8 (3.7)	136.0 (5.4)	107.4 (4.7)**
Incremental_240-min_ glucose AUC (mmol/L×240)	128.0 (4.1)	138.5 (6.1)	122.9 (5.4)**
Peak glucose (mmol/L)	6.8 (0.04)	7.2 (0.08)	6.6 (0.05)
Rise from fasting glucose (mmol/L)	1.9 (0.03)	2.0 (0.06)	1.8 (0.04)**
Decline from peak glucose (mmol/L)	−1.7 (0.04)	−2.0 (0.08)	−1.6 (0.05)**
Fasting plasma insulin (pmol/L)	241.6 (5.5)	285.1 (9.5)	219.4 (6.5)**
Total_180-min_ insulin AUC (pmol/L×180)	143694.5 (4013.1)	172487.3 (6828.7)	129036.3 (4767.5)**
Total_240-min_ insulin AUC (pmol/L×240)	164673.7 (4529.5)	198163.1 (7634.2)	147726.1 (5394.7)**
Incremental_180-min_ insulin AUC (pmol/L×180)	100311.5 (3315)	121481.6 (5718.5)	89533.9 (3944.0)**
Incremental_240-min_ insulin AUC (pmol/L×240)	106852.3 (3594.8)	130248.4 (6103.8)	95012.5 (4308.1)**
Peak insulin (pmol/L)	1483.7 (40.6)	1666.6 (64.1)	1390.3 (51.2)**
Rise from fasting insulin (pmol/L)	1241.9 (37.1)	1381.4 (58.9)	1170.7 (46.9)**
Decline from peak insulin (pmol/L)	−1181.9 (36.1)	−1291.9 (58.6)	−1126.3 (45.3)*

Values are expressed as means ± SEM or *n* (%) unless specified otherwise.

^a^Median (IQR); *p < .05, **p < 0.01. EMBS: estimated metabolic body size calculated as, fat free mass + 18 kg; AUC: area under the curve; OGTT insulin values regressed to original charcoal assay. SWIA (Southwestern Indigenous American) heritage (Full vs. Other).

### MMTT glucose AUC/iAUCs

**Fig. 2:**
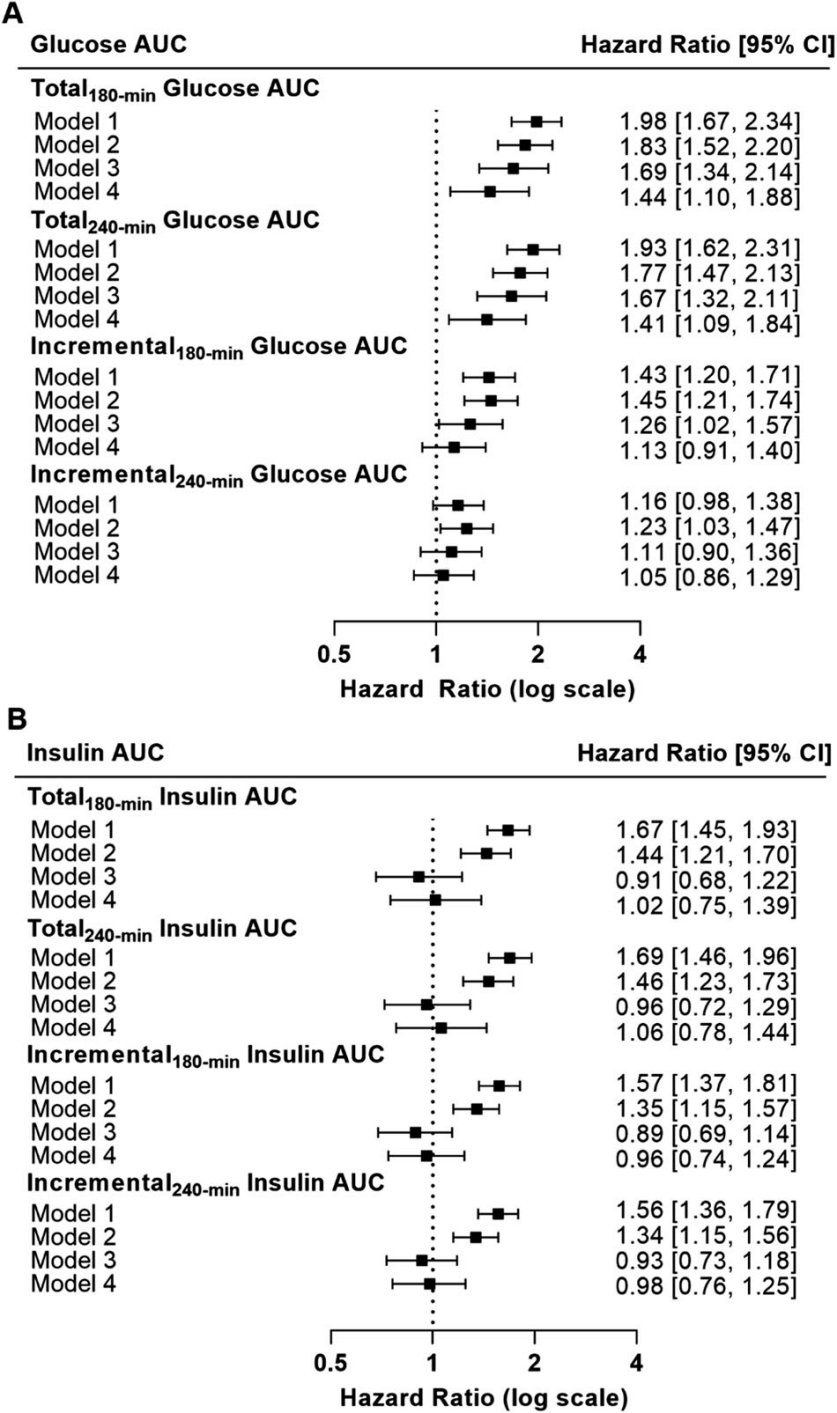
Hazard ratios (per one-SD difference) and 95% confidence intervals from Cox proportional hazard models assessing the prospective relationship between MMTT glucose (A) and insulin (B) responses on development of diabetes. Model 1 (unadjusted); Model 2 adjusted for age, sex, body fat (%); Model 3 further adjusted for M; Model 4 further adjusted for AIR and full vs. non-full Southwestern Indigenous American heritage (SWIA).

The HR and 95% confidence intervals from the Cox proportional hazards models assessing the prospective relationship between MMTT glucose AUC/iAUCs on development of diabetes are reported in Fig. 2A and Supplemental Table S1. In unadjusted analyses, glucose AUC_180-min_ (HR: 1.98, 95% CI: 1.67, 2.34, *p* < 0.0001), AUC_240-min_ (HR: 1.93, 95% CI: 1.62, 2.31, *p* < 0.0001), and iAUC_180-min_ (HR: 1.43, 95% CI: 1.20, 1.71, *p* < 0.0001) were associated with an increased risk of diabetes while iAUC_240-min_ (HR: 1.16, 95% CI: 0.98, 1.38, *p* = 0.09) was not. After further adjustment for covariates (age, sex, body fat [%], M, AIR, SWIA heritage) in three subsequent models AUC_180-min_ (model 4 HR: 1.44, 95% CI: 1.10, 1.88, *p* = 0.007) and AUC_240-min_ (model 4 HR: 1.41, 95% CI: 1.09, 1.84, *p* = 0.01) remained associated with increased risk of diabetes. In the final model, after further adjustment for AIR and SWIA, iAUC_180-min_ was no longer associated with increased risk of diabetes (HR: 1.13, 95% CI: 0.91, 1.40, *p* = 0.27). We also examined glucose and insulin AUC/iAUCs from the OGTT for comparison. These are shown in Supplement Table 3, and as expected, glucose AUC/iAUCs predicted the development of diabetes.

### MMTT insulin AUC/iAUCs

The HR and 95% confidence intervals from the Cox proportional hazards models assessing the prospective relationship between MMTT insulin AUC/iAUCs on development of diabetes are reported in Fig. 2B and Supplemental Table S2. In the unadjusted analyses and adjustment for age, sex, and body fat (%), insulin AUC_180-min_, AUC_240-min_, iAUC_180-min_, and iAUC_240-min_ were all independently associated with increased risk of diabetes (*p-*values < 0.01). However, after adjustment for M (model 3) and further adjustment for AIR and SWIA heritage (model 4) the association between insulin AUC/iAUCs with risk of diabetes was attenuated (*p-*values > 0.05). As expected, insulin AUC/iAUCs from the OGTT predicted the development of diabetes (Supplemental Table S3**)**.

### MMTT peak, rise from fasting, decline from peak

The HR and 95% confidence intervals from the Cox proportional hazards models assessing the prospective relationship between MMTT glucose/insulin peaks, rise from fasting, and decline from peak on development of diabetes are reported in Table 2. Peak glucose was consistently associated with increased risk of diabetes across all four models (model 4 HR: 1.28, 95% CI: 1.03, 1.61, *p* = 0.03). The association between glucose rise from fasting on risk of diabetes was attenuated after adjustment for AIR and SWIA heritage. Glucose decline from peak was protective against diabetes in models 1 (unadjusted), 2 and 3 but not the final model 4. Insulin peak, rise from fasting and decline from peak were not associated with the risk of diabetes after adjusting for age, sex, and body fat (%) in model 2. Results were similar using the rate of change for rise from fasting and decline from peak to adjust for time (data not shown).

**Table 2:** Hazard ratios and 95% confidence intervals of peak and absolute change (i.e. Δ) in glucose and insulin post-MMTT as predictors of type 2 diabetes.

	Peak Glucose	Rise Glucose	Decline Glucose		Peak Insulin	Rise Insulin	Decline Insulin
**Model 1**				**Model 1**			
Glucose	1.59 (1.36, 1.86)**	1.22 (1.04, 1.42)*	0.67 (0.58, 0.78)**	Insulin	1.23 (1.10, 1.38)**	1.19 (1.06, 1.34)**	0.86 (0.76, 0.98)*
**Model 2**				**Model 2**			
Glucose	1.54 (1.54, 1.82)**	1.29 (1.10, 1.52)**	0.70 (0.59, 0.82)**	Insulin	1.08 (0.95, 1.24)	1.06 (0.93, 1.21)	0.97 (0.85, 1.11)
Age	1.09 (1.09, 1.29)	1.16 (0.98, 1.36)	1.11 (0.93, 1.31)	Age	1.22 (1.05, 1.42)*	1.22 (1.05, 1.43)**	1.23 (1.06, 1.44)*
Sex	0.47 (0.47, 0.71)**	0.53 (0.35, 0.80)**	0.49 (0.33, 0.75)**	Sex	0.71 (0.50, 1.03)	0.71 (0.49, 1.02)	0.71 (0.49, 1.02)
Body Fat (%)	2.01 (2.01, 2.53)**	2.11 (1.67, 2.66)**	2.00 (1.59, 2.51)**	Body Fat (%)	1.71 (1.40, 2.10)**	1.73 (1.42, 2.12)**	1.74 (1.43, 2.12)**
**Model 3**				**Model 3**			
Glucose	1.50 (1.23, 1.84)**	1.26 (1.04, 1.53)*	0.72 (0.59, 0.87)**	Insulin	0.76 (0.62, 0.94)*	0.76 (0.62, 0.93)*	1.35 (1.10, 1.66)**
Age	0.96 (0.79, 1.18)	1.01 (0.83, 1.23)	0.97 (0.80, 1.19)	Age	1.05 (0.88, 1.27)	1.05 (0.87, 1.27)	1.05 (0.87, 1.26)
Sex	0.63 (0.36, 1.11)	0.77 (0.44, 1.33)	0.68 (0.39, 1.19)	Sex	0.79 (0.47, 1.34)	0.79 (0.46, 1.34)	0.77 (0.45, 1.30)
Body Fat (%)	1.55 (1.10, 2.17)**	1.52 (1.08, 2.14)*	1.52 (1.08, 2.12)*	Body Fat (%)	1.59 (1.15, 2.19)**	1.57 (1.14, 2.17)*	1.59 (1.15, 2.19)*
M (log)	0.58 (0.41, 0.82)**	0.53 (0.38, 0.75)**	0.56 (0.40, 0.79)**	M (log)	0.45 (0.31, 0.65)**	0.45 (0.31, 0.65)**	0.45 (0.31, 0.64)**
**Model 4**				**Model 4**			
Glucose	1.28 (1.03 1.61)*	1.11 (0.91, 1.35)	0.83 (0.67, 1.03)	Insulin	0.85 (0.68, 1.06)	0.84 (0.67, 1.03)	1.19 (0.96, 1.48)
Age	0.87 (0.70, 1.07)	0.87 (0.70, 1.07)	0.87 (0.70, 1.07)	Age	0.89 (0.73, 1.09)	0.89 (0.73, 1.09)	0.89 (0.73, 1.09)
Sex	0.63 (0.35, 1.14)	0.73 (0.41, 1.28)	0.67 (0.38, 1.20)	Sex	0.70 (0.40, 1.20)	0.69 (0.40, 1.19)	0.69 (0.40, 1.19)
Body Fat (%)	1.60 (1.14, 2.26)**	1.58 (1.12, 2.22)**	1.58 (1.13, 2.22)*	Body Fat (%)	1.68 (1.21, 2.34)**	1.68 (1.21, 2.33)**	1.68 (1.21, 2.33)**
M (log)	0.53 (0.37, 0.76)**	0.49 (0.34, 0.70)**	0.52 (0.36, 0.74)**	M (log)	0.46 (0.32, 0.67)**	0.46 (0.32, 0.66)**	0.46 (0.32, 0.67)**
AIR (log)	0.77 (0.64, 0.93)**	0.72 (0.61, 0.86)**	0.76 (0.63, 0.91)**	AIR (log)	0.69 (0.59, 0.81)**	0.69 (0.59, 0.81)**	0.70 (0.60, 0.83)**
SWIA heritage	1.79 (1.07, 3.02)*	1.87 (1.11, 3.13)*	1.86 (1.11, 3.12)*	SWIA heritage	1.80 (1.09, 2.98)*	1.80 (1.09, 2.97)*	1.78 (1.07, 2.94)*

All continuous variables were standardized (mean = 0, standard deviation = 1) and the hazard ratios are reported per standard deviation. Abbreviations include SWIA (Southwestern Indigenous American) heritage (Full vs. Other); M (insulin action for hyperinsulinemic-euglycemic clamp); AIR (acute insulin response for intravenous glucose tolerance test).

*p < 0.05.

**p < 0.01.

### DI as a predictor for MMTT and OGTT

The calculated DI using surrogate measures from the MMTT and OGTT were included in model 2. For MMTT, this measure of DI did not predict diabetes (HR: 0.91; 95% CI: 0.81, 1.03, *p* = 0.12) but did for OGTT (HR: 0.89; 95% CI: 0.81, 0.99, *p* = 0.03).

### C-Statistic comparisons of MMTT and OGTT

Correlation coefficients between MMTT and OGTT glucose variables are reported in Supplemental Table S4. Due to the moderate to strong correlations between MMTT and OGTT variables C-statistics were computed. C-statistics provide a global measure of model discrimination and were calculated to compare MMTT variables that remained significant after adjustments for age, sex, body fat (%), M, AIR, and SWIA heritage (AUC_180-min_, AUC_240-min_, and peak MMTT glucose) to the corresponding OGTT variable, fasting, 60-minute and 120-minute glucose (Supplemental Table S5). The C-statistics for MMTT AUC_180-min_, AUC_240-min_, and peak glucoses were similar to OGTT 60-min glucose (AUC_180-min_ 0.72 vs. 0.74, *p* = 0.31; AUC_240-min_ 0.71 vs. 0.74, *p* = 0.23; peak 0.71 vs. 0.74, *p* = 0.07) and OGTT 120-min glucose (AUC_180-min_ 0.72 vs 0.71, *p* = 0.86; AUC_240-min_ 0.71 vs. 0.72, *p* = 0.90; peak 0.71 vs. 0.69, *p* = 0.53). Similarly, MMTT AUC_180-min_ and AUC_240-min_ were similar to the OGTT AUC_180-min_ (AUC_180-min_ 0.72 vs. 0.74, *p* = 0.15; AUC_240-min_ 0.71 vs. 0.74, *p* = 0.90) while MMTT peak glucose were also similar to OGTT peak glucose (0.71 vs. 0.73, *p* = 0.18). The only difference occurred between MMTT glucose AUC_180-min_ and fasting glucose from the OGTT (0.72 vs. 0.69, *p* = 0.03) indicating the MMTT glucose AUC_180-min_ is slightly more predictive of diabetes compared to fasting glucose from the OGTT.

## Discussion

The aim of this analysis was to investigate the utility of an MMTT to predict incident type 2 diabetes. This study, involving a large sample in a well-defined population with detailed reference measurements of important risk factors for diabetes (e.g., insulin action, insulin secretion, and body fat), demonstrated that glucose and insulin responses to a mixed-meal challenge, predicts the development of diabetes even when accounting for these confounders.

Although the glucose and insulin curves were slightly flatter in the MMTT than the OGTT, higher glucose responses in the MMTT were still associated with later development of type 2 diabetes. Clinically, flatter curves reflect the slower carbohydrate absorption expected in a mixed meal test. This may limit the overall variability of the glucose response, but as we have shown the AUCs remain predictive of diabetes in a manner similar to OGTTs. However, in research settings there are many advantages physiologically to MMTTs, including the ability to assess more global physiologic responses including lipid metabolism which, in itself, is an important metabolic pathway for insulin resistance. Across all four models, total glucose AUCs from the MMTT were each independently associated with an increased risk of diabetes even after adjustment for insulin action and secretion. Insulin responses to the MMTT were a consistent predictor of incident diabetes. However, these associations were no longer significant after adjustment for insulin action and secretion.

While other studies have used an MMTT to evaluate glucose and insulin responses in adults with and without type 2 diabetes [3, 9, 29, 30], to our knowledge, ours is the first that has assessed whether responses to an MMTT as predictors of type 2 diabetes. Prior studies that have used an MMTT in adults have found that there is a large amount of inter-individual variability that may be accounted for by genetics, the microbiome, and a plethora of other physiological factors [4, 5, 29, 30]. In studies directly comparing MMTT and OGTTs, glucose responses generate slightly flatter curves in the MMTT compared to the OGTT [29, 30]. MMTTs generate additional and clinically useful data across a range of physiological systems in addition to the risk for type 2 diabetes, including endothelial, renal, and hepatic functioning [3]. Given this, MMTTs are used with increasing frequency in precision nutrition studies. Our data, combined with findings from others, demonstrates that in addition to assessing response to diet interventions, MMTTs also can predict important clinically relevant outcomes.

To date, there is a lack of consensus for designing a standardized MMTT. While this study employed a solid food plus liquid calorie combination, other studies have utilized varying combinations of solid foods [5], solids plus liquids [9, 30], and liquids only [29]. A strength of this study is that the MMTT design used standardized meals that were scaled to be isocaloric to a participant’s weight-maintaining energy needs, providing approximately one-third of their calorie needs for the day within a given meal. Reference measures for insulin action (M) and insulin secretion (AIR) were also included as important covariates in the analyses. MMTT AUC glucose predicted the development of diabetes independent of M and AIR indicating that the mixed macronutrient glucose responses can provide important prognostic indices above gold standard measures of insulin resistance and secretion. Our sample size was also robust; however, it was relatively homogenous as all participants were self-identified as Indigenous Americans. Therefore, it is unknown how results would differ in other populations, but since the underlying pathophysiology of type 2 diabetes is shared, we expect our results to be generalizable. We also recognize that macronutrient content and even such factors as the rate of meal or order of meal ingestion may limit the comparison of results between studies. Although we know the overall macronutrient content of the diets, that the same meals were served for the test, and the calorie load, we do not have the precise meal recipes. We realize this presents challenges in the replication of our data. However, the longitudinal follow-up in our cohort is unique so we feel our data is of value. Lastly, the authors acknowledge that the diagnosis of diabetes is based upon the OGTT rather than diabetes-specific complications such as retinopathy. Future studies may consider evaluating the comparability of the OGTT and MMTT on such clinical endpoints of type 2 diabetes (e.g., retinopathy and/or renal function).

## Conclusion

Glucose and insulin responses to body size-adjusted mixed macronutrient challenges predicted the development of type 2 diabetes. Our data indicate that MMTT response. which simulates more “real world” intake can inform health risks beyond the immediate response to dietary interventions.

### Supplementary information

Supplemental Material

## Data Availability

The datasets generated during and/or analyzed during the current study are not publicly available due to previous agreements made between the NIDDK and Indigenous nations who participated in this study. Inquiries may be made to the corresponding author.
