# Supplementary material for: A mixed meal tolerance test predicts onset of type 2 diabetes in Southwestern Indigenous adults
Source: Nutr Diabetes. 2024 Jul 10;14:50. doi: 10.1038/s41387-024-00269-3 (PMC11237083; doi:10.1038/s41387-024-00269-3)
Supplement: Supplementary file 1 — Supplemental Material [file 41387_2024_269_MOESM1_ESM.docx]

# Supplemental Material

**Supplemental Table S1.**

| **Supplemental Table S1. Hazard Ratios and 95% Confidence Intervals of the Association Between MMTT Glucose AUC/iAUCs and Type 2 Diabetes** | | | | |
| --- | --- | --- | --- | --- |
|  | AUC_180-min_ | AUC_240-min_ | iAUC_180-min_ | iAUC_240-min_ |
| **Model 1** | | | | |
| Glucose | 1.98 (1.67, 2.34)** | 1.93 (1.62, 2.31)** | 1.43 (1.20, 1.71)** | 1.16 (0.98, 1.38) |
| **Model 2** | | | | |
| Glucose | 1.83 (1.52, 2.20)** | 1.77 (1.47, 2.13)** | 1.45 (1.21, 1.74)** | 1.23 (1.03, 1.47)* |
| Age | 1.04 (0.87, 1.23) | 1.07 (0.90, 1.26) | 1.14 (0.97, 1.34) | 1.18 (1.00, 1.39)* |
| Sex | 0.49 (0.33, 0.75)** | 0.49 (0.32, 0.73)** | 0.60 (0.40, 0.89)** | 0.60 (0.40, 0.88)** |
| Body Fat (%) | 1.78 (1.42, 2.23)** | 1.82 (1.45, 2.27)** | 2.00 (1.60, 2.51)** | 2.02 (1.61, 2.53)** |
| **Model 3** | | | | |
| Glucose | 1.69 (1.34, 2.14)** | 1.67 (1.32, 2.11)** | 1.26 (1.02, 1.57)* | 1.11 (0.90, 1.36) |
| Age | 0.93 (0.76, 1.14) | 0.95 (0.78, 1.16) | 1.01 (0.83, 1.23) | 1.04 (0.85, 1.26) |
| Sex | 0.66 (0.37, 1.15) | 0.65 (0.37, 1.14)* | 0.86 (0.50, 1.48) | 0.86 (0.50, 1.48) |
| Body Fat (%) | 1.44 (1.03, 2.01)** | 1.45 (1.04, 2.02)** | 1.42 (1.02, 1.99)* | 1.42 (1.02, 1.98)* |
| M (log) | 0.63 (0.44, 0.89)** | 0.62 (0.44, 0.87)** | 0.54 (0.38, 0.77)** | 0.52 (0.37, 0.73)** |
| **Model 4** | | | | |
| Glucose | 1.44 (1.10, 1.88)** | 1.41 (1.09, 1.84)** | 1.13 (0.91, 1.40) | 1.05 (0.86, 1.29) |
| Age | 0.85 (0.69, 1.06) | 0.86 (0.69, 1.06) | 0.86 (0.70, 1.07) | 0.87 (0.70, 1.08) |
| Sex | 0.64 (0.36, 1.14) | 0.63 (0.36, 1.13) | 0.78 (0.44, 1.36) | 0.76 (0.44, 1.33) |
| Body Fat (%) | 1.53 (1.09, 2.15)** | 1.54 (1.10, 2.17)** | 1.53 (1.09, 2.15)** | 1.55 (1.10, 2.17)** |
| M (log) | 0.58 (0.40, 0.85)** | 0.57 (0.39, 0.82)** | 0.50 (0.35, 0.72)** | 0.49 (0.34, 0.70)** |
| AIR (log) | 0.81 (0.67, 0.98)* | 0.79 (0.66, 0.95)** | 0.73 (0.61, 0.86)** | 0.70 (0.60, 0.83)** |
| SWIA heritage | 1.73 (1.03, 2.92)* | 1.74 (1.03, 2.93)* | 1.85 (1.11, 3.11)* | 1.89 (1.13, 3.17)* |
| *All continuous variables were standardized (mean=0, standard deviation=1) and the hazard ratios are reported per standard deviation. Abbreviations include: SWIA (Southwestern Indigenous American) heritage (Full vs. Other); M (insulin action for hyperinsulinemic-euglycemic clamp); AIR (acute insulin response for intravenous glucose tolerance test).*p<0.05; **p<0.01* | | | | |

**Supplemental Table S2.**

| **Supplemental Table S2. Hazard Ratios and 95% Confidence Intervals of the Association Between MMTT Insulin AUC/iAUCs and Type 2 Diabetes** | | | | |
| --- | --- | --- | --- | --- |
|  | AUC_180-min_ | AUC_240-min_ | iAUC_180-min_ | iAUC_240-min_ |
| **Model 1** | | | | |
| Insulin† | 1.67 (1.45 , 1.93)** | 1.69 (1.46, 1.96)** | 1.57 (1.37, 1.81)** | 1.56 (1.36, 1.79)** |
| **Model 2** | | | | |
| Insulin† | 1.44 (1.21, 1.70)** | 1.46 (1.23, 1.73)** | 1.35 (1.15, 1.57)** | 1.34 (1.15, 1.56)** |
| Age | 1.18 (1.01, 1.37)* | 1.18 (1.01, 1.38)* | 1.18 (1.01, 1.38)* | 1.19 (1.02, 1.39)* |
| Sex | 0.77 (0.53, 1.11) | 0.75 (0.52, 1.08) | 0.75 (0.52, 1.08) | 0.73 (0.50, 1.05) |
| Body Fat (%) | 1.44 (1.15, 1.81)** | 1.43 (1.15, 1.80)** | 1.53 (1.27, 1.90)** | 1.54 (1.25, 1.91)** |
| **Model 3** |  |  |  |  |
| Insulin† | 0.91 (0.68, 1.22) | 0.96 (0.72, 1.29) | 0.89 (0.69, 1.14) | 0.93 (0.73, 1.18) |
| Age | 1.07 (0.89, 1.29) | 1.07 (0.89, 1.29) | 1.07 (0.89, 1.29) | 1.07 (0.89, 1.29) |
| Sex | 0.88 (0.52, 1.49) | 0.86 (0.51, 1.46) | 0.88 (0.52, 1.49) | 0.86 (0.51, 1.45) |
| Body Fat (%) | 1.51 (1.08, 2.11)* | 1.49 (1.06, 2.08)* | 1.51 (1.09, 2.10)** | 1.50 (1.08, 2.08)* |
| M (log) | 0.52 (0.35, 0.78)** | 0.54 (0.36, 0.80)** | 0.51 (0.35, 0.75)** | 0.52 (0.36, 0.77)** |
| **Model 4** | | | | |
| Insulin† | 1.02 (0.75, 1.39) | 1.06 (0.78, 1.44) | 0.96 (0.74, 1.24) | 0.98 (0.76, 1.25) |
| Age | 0.88 (0.72, 1.09) | 0.88 (0.71, 1.08) | 0.89 (0.73, 1.09) | 0.88 (0.72, 1.09) |
| Sex | 0.77 (0.45, 1.32) | 0.75 (0.44, 1.29) | 0.76 (0.44, 1.30) | 0.75 (0.44, 1.27) |
| Body Fat (%) | 1.58 (1.12, 2.23)** | 1.57 (1.12, 2.22)** | 1.61 (1.15, 2.26)** | 1.62 (1.26, 2.26)** |
| M (log) | 0.53 (0.35, 0.81)** | 0.54 (0.36, 0.83)** | 0.51 (0.34, 0.75)** | 0.51 (0.34, 0.75)** |
| AIR (log) | 0.68 (0.58, 0.79)** | 0.68 (0.58, 0.79)** | 0.68 (0.59, 0.80)** | 0.68 (0.58, 0.79)** |
| SWIA heritage | 1.84 (1.11, 3.04)* | 1.85 (1.12, 3.06)* | 1.84 (1.11, 3.03)* | 1.85 (1.12, 3.06)* |
| *All continuous variables were standardized (mean=0, standard deviation=1) and the hazard ratios are reported per standard deviation. Abbreviations include: SWIA (Southwestern Indigenous American) heritage (Full vs. Other); M (insulin action for hyperinsulinemic-euglycemic clamp); AIR (acute insulin response for intravenous glucose tolerance test). † Insulin AUC/iAUCs were log transformed. *p<0.05; **p<0.01* | | | | |

**Supplemental Table S3**

| **Supplemental Table S3. Hazard Ratios and 95% Confidence Intervals of the Association Between OGTT Glucose and Insulin AUC/iAUC and Type 2 Diabetes** | | | | | |
| --- | --- | --- | --- | --- | --- |
|  | AUC_180-min_ | iAUC_180-min_ |  | AUC_180-min_ | iAUC_180-min_ |
| **Model 1** | | | **Model 1** | | |
| Glucose | 2.08 (1.78, 2.43)** | 1.84 (1.58, 2.15)** | Insulin | 1.35 (1.20, 1.51)** | 1.31 (1.16, 1.47)* |
| **Model 2** | | | **Model 2** | | |
| Glucose | 2.05 (1.73, 2.43)** | 1.81 (1.54, 2.13)** | Insulin | 1.19 (1.04, 1.36)* | 1.16 (1.02, 1.33)* |
| Age | 1.05 (0.89, 1.24) | 1.11 (0.95, 1.31) | Age | 1.22 (1.04, 1.42)* | 1.22 (1.05, 1.42)* |
| Sex | 0.47 (0.32, 0.70)** | 0.55 (0.38, 0.79)** | Sex | 0.71 (0.49, 1.02) | 0.70 (0.49, 1.01) |
| Body Fat (%) | 1.69 (1.39, 2.07)** | 1.80 (1.47, 2.19)** | Body Fat (%) | 1.65 (1.35, 2.03)** | 1.68 (1.37, 2.06)** |
| **Model 3** | | | **Model 3** | | |
| Glucose | 1.79 (1.44, 2.21)** | 1.59 (1.30, 1.94)** | Insulin | 0.88 (0.71, 1.10) | 0.87 (0.70, 1.08) |
| Age | 0.98 (0.81, 1.19) | 1.01 (0.84, 1.22) | Age | 1.06 (0.88, 1.28) | 1.07 (0.88, 1.28) |
| Sex | 0.56 (0.32, 0.98)* | 0.67 (0.39, 1.15) | Sex | 0.88 (0.52, 1.49) | 0.88 (0.52, 1.49) |
| Body Fat (%) | 1.61 (1.16, 2.25)* | 1.60 (1.15, 2.22)* | Body Fat (%) | 1.51 (1.09, 2.09)* | 1.51 (1.09, 2.08)* |
| M (log) | 0.72 (0.52, 1.00) | 0.64 (0.46, 0.90) | M (log) | 0.49 (0.34, 0.72)** | 0.49 (0.34, 0.71)** |
| **Model 4** | | | **Model 4** | | |
| Glucose | 1.59 (1.26, 2.00)** | 1.43 (1.15, 1.78)** | Insulin | 0.96 (0.76, 1.21) | 0.95 (0.76, 1.19) |
| Age | 0.86 (0.70, 1.06) | 0.86 (0.70, 1.06) | Age | 0.87 (0.71, 1.07) | 0.87 (0.71, 1.07) |
| Sex | 0.54 (0.30, 0.95)* | 0.63 (0.37, 1.09) | Sex | 0.75 (0.44, 1.29) | 0.75 (0.44, 1.29) |
| Body Fat (%) | 1.69 (1.21, 2.36)** | 1.66 (1.19, 2.31)** | Body Fat (%) | 1.63 (1.17, 2.28)** | 1.63 (1.17, 2.27)** |
| M (log) | 0.66 (0.47, 0.94)* | 0.60 (0.42, 0.84) | M (log) | 0.49 (0.33, 0.72)** | 0.49 (0.33, 0.71)** |
| AIR (log) | 0.76 (0.64, 0.90)** | 0.75 (0.64, 0.89)** | AIR (log) | 0.67 (0.58, 0.79)** | 0.67 (0.58, 0.79)** |
| SWIA heritage | 1.74 (1.05, 2.88)* | 1.87 (1.13, 3.08)* | SWIA | 1.82 (1.10, 3.02)* | 1.83 (1.10, 3.02)* |
| *All continuous variables were standardized (mean=0, standard deviation=1) and the hazard ratios are reported per standard deviation. Abbreviations include: SWIA (Southwestern Indigenous American) heritage (Full vs. Other); M (insulin action for hyperinsulinemic-euglycemic clamp); AIR (acute insulin response for intravenous glucose tolerance test). *p<0.05; **p<0.01* | | | | | |

| **Supplemental Table S4.** Correlation Matrix of Glucose MMTT and OGTT Variables | | | | | | | | |
| --- | --- | --- | --- | --- | --- | --- | --- | --- |
|  | **1** | **2** | **3** | **4** | **5** | **6** | **7** | **8** |
| 1. MMTT AUC_180-min_ | -- |  |  |  |  |  |  |  |
| 2. MMTT AUC_240-min_ | **0.98** | -- |  |  |  |  |  |  |
| 3. MMTT Peak | **0.81** | **0.77** | -- |  |  |  |  |  |
| 4. OGTT Fasting | **0.62** | **0.63** | **0.51** | -- |  |  |  |  |
| 5. OGTT 60-minute | **0.50** | **0.44** | **0.50** | **0.43** | -- |  |  |  |
| 6. OGTT 120-minute | **0.55** | **0.52** | **0.41** | **0.43** | **0.62** | -- |  |  |
| 7. OGTT AUC_180-min_ | **0.61** | **0.56** | **0.51** | **0.54** | **0.86** | **0.88** | -- |  |
| 8. OGTT Peak | **0.49** | **0.43** | **0.47** | **0.41** | **0.91** | **0.61** | **0.85** | -- |
| Bolded coefficients denote statistical significance (p<0.05) | | | | | | | | |

| **Supplemental Table S5. Discriminatory properties of MMTT and OGTT glucose and insulin responses to predict Type 2 Diabetes** | | | | |
| --- | --- | --- | --- | --- |
| **Model 1** | **C-statistic (95% CI)** | **Model 2** | **C-statistic (95% CI)** | **p-value** |
| MMTT Glucose AUC_180-min_ | 0.72 (0.68, 0.76) | OGTT Fasting Glucose | 0.69 (0.64, 0.73) | 0.03 |
|  |  | OGTT 60-min Glucose | 0.74 (0.70, 0.77) | 0.31 |
|  |  | OGTT 120-minute Glucose | 0.71 (0.68, 0.75) | 0.86 |
|  |  | OGTT Glucose AUC_180-min_ | 0.74 (0.70, 0.78) | 0.15 |
| MMTT Glucose AUC_240-min_ | 0.71 (0.67, 0.75) | OGTT Fasting Glucose | 0.69 (0.65, 0.73) | 0.07 |
|  |  | OGTT 60-min Glucose | 0.74 (0.70, 0.77) | 0.23 |
|  |  | OGTT 120-minute Glucose | 0.72 (0.68, 0.75) | 0.90 |
|  |  | OGTT Glucose AUC_180-min_ | 0.74 (0.71, 0.78) | 0.09 |
| MMTT Peak Glucose | 0.71 (0.66, 0.75) | OGTT Fasting Glucose | 0.69 (0.65, 0.73) | 0.27 |
|  |  | OGTT 60-min Glucose | 0.74 (0.70, 0.77) | 0.07 |
|  |  | OGTT 120-minute Glucose | 0.72 (0.68, 0.76) | 0.53 |
|  |  | OGTT Peak Glucose | 0.73 (0.69, 0.76) | 0.18 |
| C-statistics were calculated from models accounting for time-to-event and are analogous to the area under the ROC curve. P-values indicate significance of the difference in C-statistic from that of Model 1. Data are reported as C-statistic (95% CI, confidence interval). All models were adjusted for covariates (age, sex, body (%), M (insulin action for hyperinsulinemic-euglycemic clamp), AIR (acute insulin response for intravenous glucose tolerance test), and SWIA (Southwestern Indigenous American) heritage). | | | | |
